# Anatomical and Physiological Relations of the Mouth

**Published:** 1848-04

**Authors:** W. R. Handy

**Affiliations:** Baltimore.


### ARTICLE X.

*Anatomical and Physiological Relations of the Mouth.* By Professor W. R. HANDY, M. D., Baltimore.

The surgeon dentist claims the *mouth* as his especial province—the particular field of all his labors—and the constant exercise and triumph of his art.

The natural inference from a statement like this, would be, that such an individual must have the most complete knowledge of the mouth, and the most thorough mastery of it in every particular over that of every one else—such should be the fact—but is such the true state of the case? Does it, we venture to ask, even approach to any thing like the truth in the matter? On the contrary, have we not most sadly to deplore, and most keenly to feel, on behalf of suffering humanity, the very, yes, we repeat it, the very *limited knowledge* even of the mouth, possessed by the great mass of those who pretend to practice dentistry?

Without any intention, whatever, to give offence, we most honestly ask, how many out of the great body of self constituted dentists, scattered over the length and breadth of our country, have a general, much less a precise, knowledge of the dental organs? their structure, functions, relations among themselves, and with the system at large?

The answer must certainly be, that it is only the few that possess any scientific acquaintance with the mouth, while the great bulk, it must be acknowledged, view the mouth as little more than a blacksmith's shop, where they as mechanically extract a tooth and adjust a plate, as the smith fits the shoe and drives the nail to the horse's foot.

But we are happy to be able to state, that the dental profession, students of dentistry, and even the people at large, are beginning to be awakened to the importance of this subject—to wit: the absolute necessity of a scientific dental education—and as one of the fundamental and indispensable elements to be taught in such a system, the study of anatomy and physiology—the alarm has been sounded, and the little Spartan band of dental heroes who have long painfully felt that their profession should have the same dignity and elevation of the medical; that as each were part and parcel of the same great healing art—that, consequently, each should be placed on the same broad equality; to stand side by side, and share alike in equal honors and esteem and equal trustworthiness and confidence from the community. We say, that this noble little band, who have so long struggled for such a result, are now beginning to realise the fruit of their hard earned toil; are now beginning to see the dawn of the future meridian sun, when in all the splendor of his brightness, he will make the circuit of his course, side by side, with that of his medical brother; that the day is fast approaching when the science of dentistry will be acknowledged to have the same family origin as that of medicine; when the claims of relationship will not only be established between the two, but be gladly embraced by both; and when the twin brothers so long divided, will now be seen to be walking hand in hand, and marching joyfully together, onward and upward, each in their respective spheres, though both in the same great circle of good—striving alike to reach the goal of their utmost ambition, to wit: the blessing of mankind, by removing disease, restoring health, and thus augmenting the amount of human health and happiness.

To hasten such a desirable result, and keep alive the attention already awakened to the subject, we propose in the present article, not only to still further direct attention to, but to convert, if possible, by our feeble efforts, this now aroused attention, into a deep, thorough, and abiding conviction, and into a firm, well settled, and unshaken principle of future thought and action.

To do this, we shall attempt an examination of the relations of the *mouth*, and enter into a somewhat critical analysis of its anatomical and physiological relations, as furnishing one of the strongest arguments, why every dentist should study anatomy as a fundamental and essential means to the attainment of skill, success and eminence in his profession; and without which, there must be certain failure in greater or less degree in all these great requisites.

The relations of the mouth in their most comprehensive sense, are co-extensive with the whole being of man—physical, organic and mental.

By its physical relations, the mouth is made the medium of the air we breathe, and of the food and drinks we take to nourish our bodies.

By the organic, it is united to all the organs of the body, thus forming an essential link in that great and mysterious chain, the various parts of which are bound together in one great brotherhood; forming thereby one common sympathy, one common interest, and one common enjoyment among the whole family of organs.

And by its mental relations, the mouth is the instrument of music, speech and oratory.

It is the organic relations of the mouth, which chiefly claim our attention; and these are the anatomical and physiological.

The anatomical elements of the mouth, are the same as those seen in every part of the body—we here find bone, muscle, gland, blood-vessel, nerve, cellular, mucous structures, and all entering as elements in its formation. These elements variously combined constitute the dental organs, and the varied actions of these organs comprise the functions of the dental apparatus. Now these same elements, with some others, though differently combined, enter into and form all the other organs of the body with all their wonderful variety in form, size, color, density, structure and function; and further, many of these same elements of the mouth can be easily and distinctly traced into other and distant organs, and, in fact, with some throughout the whole system.

The mucous membrane of the mouth can be readily followed by one direction into the pharynx, oseophagus, stomach, intestines and collatitious organs, through their excretory ducts; and from the same point, as readily traced in another direction into the larynx, trachea, bronchia and lungs; thus, not only connecting all these various and remote organs, by one continuous anatomical structure; but further, by combining its functions with those of these several organs, of thus entering into and forming a most essential element in the complicated process of digestion and respiration—processes whose end is no less than the conversion of our food, first into chyme and chyle, and finally, into blood—the vital pabulum of formation, growth, and sustentation to the whole body.

The blood-vessels of the mouth, to wit: the arteries can be traced back to the external carotid, which, uniting with the internal carotid artery, forms the common carotid, which is traced still further back into the arteria-innominata of the right side and arch of the aorta on the left, and both ultimately into the heart; the great centre of the circulation and the common source from whence the blood takes its departure, to be distributed throughout the whole system; thus it is seen, that these two elements, simply of the mouth, the mucous membrane and its arteries, enter most largely into the whole economy, and connect functions at once the most varied, complicated and vital.

The *nerves* of the mouth can be traced to the brain and spinal marrow, thus uniting it with the sources of thought and voluntary power; and thereby putting it in the most intimate relation with all the organs to which the nerves, emanating from these quarters, are distributed, together with all their functions; comprising all the organs of relation, those which connect us with the external world, and consisting of locomotion, speech, and sense.

The glosso-pharyngeal and parvagum of the eighth pair of nerves, is the direct route of connection between the mouth, brain, pharynx, oseophagus, lungs and stomach ; while the frequent anastomosis of the sympathetic, with these same nerves, establishes still more closely and extensively the inseparable relation which the mouth has both with the organs of relation and nutrition.

That the relation of the mouth, is thus intimate, extensive and direct with these two great classes of organs, it is only necessary to mark the effect when any disturbance in their physiology or healthy action occurs.

Suppose, for instance, the brain becomes injured, or some most sudden, unexpected and disastrous news reaches it, immediately the eighth pair of nerves, with telegraphic speed, conveys the impression to the mouth, lungs and stomach ; and as quick as thought, the healthy action of these organs will be more or less interrupted ; the stomach, let its appetite be ever so keen, will have an immediate loathing for food, and if at dinner, will at once, most probably, vomit what had been taken.

The lungs will almost instantly cease to breathe, while the countenance is pale or otherwise, as the blood circulates or not, in the face.

Or let a child have difficult dentition, and the irritation of the gums, through the fifth pair of nerves, will be extended to the brain, and from thence be reflected upon the muscular system, producing convulsions, frequently, both frightful and dangerous : thus illustrating the relation of the mouth with the brain and muscular organs.

The cellular membrane of the mouth is *universal* with the whole body ; it enters as an essential element in the constitution of every organ ; surrounds, separates and connects every part, and has been beautifully compared to a mould, in which every organ receives its original and proper shape.

The *muscles* of the mouth possess the same physical and vital properties with the rest of the muscular system ; so that in cases of general or partial paralysis, the mouth often partakes in the same affection, and may be either drawn to the one side, as in hemiplegia, or completely and firmly closed as in lock-jaw.

The *glands* of the mouth, forming another of its elements, and consisting principally of mucous follicles, are continuous with the same extensive chain, found strewed throughout the whole alimentary tube, from one end to the other.

*Pathology* might here be invoked to establish, if possible, still more conclusively this general relationship of the mouth with the system at large; for when the organs are in perfect health, each performing its appropriate function, and the whole acting in the utmost harmony, in the great drama of life—we say it is not always in such a state of things, that the general relations of the body are most readily detected and traced. On the contrary, it is often necessary that the discord of disease should enter into this happy family of organs, and disturb the mutual understanding, and constant and repeated acts of friendship and kindness; with all the reciprocal and mutual benefits, unceasingly flowing from such relationship—we say it is only then, that we become fully sensible, how deep is the bond of sympathy which unites each organ to every other organ, and how strong the ties which bind the whole to every part and every part to the whole; thus beautifully illustrating the mutual fellowship, dependancy and assistance which each most cordially extends to the other and the whole to each.

As for instance, fever is a general disease, and attacks the whole system. Do we find the mouth idle in this general assault any more than any other part? On the contrary, do we not find by a variety of the most significant expressions, as dryness of the mouth, furred tongue, parched lips, that it not only has a close family relationship with the system at large, but proves it, practically, by both feeling and sharing largely in the general distress, and endeavoring as far as possible to divide the calamity, common to the whole?

In scurvy, another constitutional disease, the *gums* swell, become spongy and bleed; thus showing that they sympathise with and partake of the relaxed and debilitated condition belonging to the whole body; and so with a variety of other examples unnecessary to cite, where this general relation between the mouth and rest of the system is equally shown.

Indeed, so close is this relation between the mouth and the body generally, and so vastly important is it considered by the medical profession, and being so viewed from time immemorial, that every physician invariably examines the mouth and especially the tongue, to ascertain the general condition and health of the system.

In fact, thus regarding the mouth as the *prime sentinel* of the many stationed by nature upon the watch tower of the body, whose duty it is to give accurate report of any changes occurring in the system, and to faithfully sound the alarm when there is any departure in the healthy action of the several organs.

It may be necessary now, to say a word upon the relation existing between the different organs and functions comprising the mouth itself, which may be styled its special or domestic relations.

That such relations exist, seem almost self-evident, and also as infallibly certain, that if any thing should occur to derange these relations, discord and confusion will be the necessary result; in the language of the proverb, "a house divided against itself must fall."

The mouth, as already stated, consists of a variety of organs and equally as great a variety of functions. It contains the organs of prehension, mastication and insalivation; also, the additional functions of suction, speech and expression; with the special sense of taste, and the general sense of feeling.

Now if any of these organs or functions be impaired or lost, there is more or less disturbance among all the rest.

Who is not familiar how the loss of the teeth, a cleft palate, and swollen tonsils alters the speech, changes the voice, and renders articulation frequently very difficult, if not impossible, while, at the same time, mastication is exceedingly tedious and imperfect? &c. And if the taste and general sensibility of the mouth be destroyed, all its functions concerned in the first stages of digestion will be performed, according to the experiments of Sir Charles Bell, in a slow, indifferent and irregular manner.

Enough has now been said we trust, to prove most conclusively, the first part of our proposition, to wit: that the mouth is not simply an isolated part of the body, but, on the contrary, that it has the most extensive, direct and important *anatomical* and physiological relations with the whole system.

It may here be admitted, that these relations of the mouth are all as intimate and extensive as stated, but some of our readers gravely ask the question, suppose such to be the fact, of what use is all this to the dentist? or in what conceivable way has it any thing to do, or by any possibility afford any assistance in the practice of his profession? The dentist has only to do with the mouth, if he understands that, where is the use to go further? The answer to this question brings us to the second part of our subject, which is to show that a *knowledge* of the *anatomical and physiological* relations of the mouth are essential to the scientific dentist, and that such knowledge is also equally essential to skill, success and honest eminence, in the practice of the simply mechanical dentist.

The importance of anatomy and physiology to the surgeon and physician is universally acknowledged, it is regarded as the foundation of the whole superstructure of medical and surgical science, and the only solid basis of correct medical and surgical practice; hence its deep and thorough study is every where considered and required as an essential requisite to every candidate in obtaining his degree for medical honors.

Why, and how is such knowledge so indispensable to the surgeon and physician?

We reply, that to the surgeon it tells him *when*, and *where*, and how to operate; in other words, anatomy furnishes the principles, and lays down the rules, which are to guide him in his operations, and all that the surgeon has to do is to apply those principles—hence anatomical and physiological principles are the scientific, and the only trustworthy basis of the whole surgical art and practice. A few examples may suffice in illustration.

A surgeon is called to a case of compound comminuted fracture of one of the limbs, the soft parts are dreadfully lacerated, the bones crushed into many pieces, the arteries most of them ruptured and destroyed, and the mangled limb frightfully swollen. What is to be done? are the broken fragments simply adjusted with splints and other mechanical appliances, and the case left? certainly not, for here is a problem presented to the mind, the practical solution of which calls in requisition all the resources, all the knowledge, and all the experience, however extensive it may be, that can be possibly brought to bear upon it.

For, in the first place, before a single step can be taken, the question must at once be settled? can the limb be saved or must it come off?

To answer this question satisfactorily and safely, all the anatomical and physiological knowledge of the surgeon is demanded, at the very threshhold, and at once, as a few minutes delay might be fatal.

It involves the question, what are the resources of the system? or what can nature do herself in the case, towards saving the limb?

The scientific surgeon will at once ascertain the condition of the arteries, how many are destroyed? what ones are they? the principal or secondary branches? if the principal, are the collateral branches sufficient to carry on the circulation? If it be decided they are sufficient, this is one very important item of evidence in the case, but not by any means enough to decide the question.

If the arteries be still in abundance to carry on the circulation in the limb, is it also equally certain, that the general health of the system, the digestive and blood-making organs are in a condition and will be able to manufacture such blood as will maintain the integrity of the limb, and prevent mortification?

To settle this point, a knowledge of the organs and functions of digestion, respiration, secretion in their healthy state, and, in a word, the anatomy and physiology of the whole of nutrition is necessary.

The general health of the organs being deemed adequate, the surgeon is justified in an effort to save the limb.

But on the other hand, suppose these favorable conditions on the part of the limb and the health of the system at large be wanting, then the limb must come off; the question now is, how should it be done? the answer plainly is, that anatomy is our only guide, it alone truly informs us what are the muscles to cut through, where are the arteries to be secured, what nerves to shun, in a word how the whole is to be placed in the best possible condition for being restored to health.

In the cases of hemorrhoids and fistula in ano, the surgeon has his anatomical and physiological knowledge equally taxed, and equally necessary in determining the when, and where, and how, or whether he should operate at all or not for the removal of these diseases.

If the patient be laboring under *phthisis*, where the powers of the system are broken up, or the functions of nutrition be laboring under any other grave disease, the decision of the surgeon would be rather against an operation in either case, for the reason that nature can render no assistance whatever, and that her powers, already nearly exhausted, would only be still further enfeebled, if not entirely destroyed by an operation.

To the *physician*, anatomy and physiology is equally necessary in pointing out to him the seat of the disease, the organ affected, and the nature and extent of that affection, a first and fundamental element of knowledge to the proper remedial treatment. And so with nearly the whole catalogue of surgical and medical diseases, a knowledge of their nature and seat, and the skill and success in their treatment, is, in a very large measure, due to *anatomy* and *physiology*.

Now the *mouth;* the special department of the dentist, is equally liable to accident and disease, with that of any other portions of the body, hence equally belonging to the dominion of surgery and medicine, and equally requiring the knowledge and skill of both surgeon and physician for their proper treatment; for example, the surgeon dentist is applied to for a set of artificial teeth—on examining the mouth, he finds the gums and palate inflamed, swollen, spongy and bleeding. Does he commence at once taking the impression and adjusting the plate under such circumstances? Certainly not, for the greatest ignorance as well as suffering of the patient would not tolerate so gross a violation of common sense.

Before he can do any thing either safely, skilfully or successfully, he must answer at the bar of his conscience as an honest man the following or similar questions in reference to the case. Is the inflammation common or specific, one of an ordinary character and soon subsiding, or one that is constitutional and extending deep and far back in the general system? or is it a malignant tumor, defying any or all kinds of treatment whatever?

Now the answer to these questions settles the all important point, whether it will be proper at all to apply any plate, and if proper, the time when it can be most safely done. And this answer can only be arrived at by a knowledge of the healthy structure and functions of these parts and their relations with the general system, in other words the anatomy and physiology of the mouth, and of the whole body, is necessary to form a safe and correct judgment in the matter.

For this state of the gums and palate, just described, are only so many morbid conditions or departures from the standard of health natural to these organs, hence to restore them back to this standard, it is plainly to be seen, that we must first know what that standard is.

Now the *health* of the mouth as well as every other portion of the body, depends upon the state of the general system, and this state of the general health, whether good or bad, determines the point of how soon the operation may be undertaken, for if the patient be laboring under syphilis or scurvy, these general diseases will be reflected with greater or less violence upon the mouth, and it will invariably suffer with other parts, in the precise proportion of its relation, established by the universal law of dependency, which it has with the neighboring organs, and with the whole system.

But how is the dentist to judge of the influence of disease of the body upon the mouth, when he knows nothing of the organs and functions of that body in the state of health? of their mutual action and reaction for weal or woe? And that in proportion as the organs of the body depart from their healthy standard of action, in like proportion, may we expect the

*mouth* to sympathise with and suffer in the general disturbance; and not until this general disturbance is lessened or removed, can we expect any amelioration or subsidence in the diseases of the mouth.

Hence, in the cases before us, the well informed dentist in anatomy and pathology, would know that scurvy and syphilis required constitutional treatment; that these diseases must first be eradicated, and the general health of the system first be restored before he can with any kind of propriety and success, commence the special application of his art in applying artificial teeth.

Again, without this general knowledge of anatomy and physiology, and of the reciprocal influence which the whole body and its several parts exert each upon the other, both in health and disease, it is equally impossible that the dentist can have any conceivable conception of the immense amount of injury that purely local diseases of the dental organs can produce upon the general health, and consequently how much suffering he might relieve were he in possession of this *anatomical and physiological knowledge*, which we are contending for as absolutely essential to a scientific and honest dentist.

But again and lastly, would any dentist desire to be ignorant, or to be considered ignorant of the different parts composing the mouth, their respective relations, mode of formation, development and peculiarities in contrast with the rest of the body? certainly not. But how can he possibly escape such a dilemma, when he knows nothing of the source from whence the materials for building up the dental apparatus are derived? to obtain which knowledge, the study of anatomy and physiology is his only hope.

